# The transcriptome of the NZ endemic sea urchin Kina (*Evechinus chloroticus*)

**DOI:** 10.1186/1471-2164-15-45

**Published:** 2014-01-20

**Authors:** Gareth B Gillard, Daniel J Garama, Chris M Brown

**Affiliations:** 1 Biochemistry Department, University of Otago, Dunedin, New Zealand

## Abstract

**Background:**

Sea urchins are studied as model organisms for developmental and systems biology and also produce highly valued food products. *Evechinus chloroticus* (Kina) is a sea urchin species that is indigenous to New Zealand. It is the type member of the *Evechinus* genus based on its morphological characteristics. Previous research has focused on identifying physical factors affecting commercial roe quality of *E. chloroticus*, but there is almost no genetic information available for *E. chloroticus. E. chloroticus* is the only species in its genus and has yet to be subject to molecular phylogenetic analysis.

**Results:**

In this study we performed a *de novo* transcriptome assembly of Illumina sequencing data. A total of 123 million 100 base length paired-end reads were generated using RNA-Seq libraries from a range of *E. chloroticus* tissues from two individuals obtained from Fiordland, New Zealand. The assembly resulted in a set of 75,002 transcripts with an accepted read coverage and length, of which 24,655 transcripts could be functionally annotated using protein similarity. Transcripts could be further annotated with Gene Ontology, KEGG Orthology and InterPro terms. With this sequence data we could perform the first phylogenetic analysis of *E. chloroticus* to other species of its family using multiple genes. When sequences for the mitochondrial nitrogen dehydrogenase genes were compared, *E. chloroticus* remained outside of a family level clade, which indicated *E. chloroticus* is indeed a genetically distinct genus within its family.

**Conclusions:**

This study has produced a large set of *E. chloroticus* transcripts/proteins along with functional annotations, vastly increasing the amount of genomic data available for this species. This provides a resource for current and future studies on *E. chloroticus*, either to increase its commercial value, or its use as a model organism. The phylogenetic results provide a basis for further analysis of relationships between *E. chloroticus*, its family members, and its evolutionary history.

## Background

New Zealand coastal zones contain an abundant population of the native sea urchin species *Evechinus chloroticus*, locally known by the Maori name, Kina. It is the type member of its genus, but there has been little molecular analysis performed on this species to date. Sea urchin roe (gonads of male and female sea urchins) is a highly valued food product internationally, with the largest demand coming from Japan where the roe, locally known as “uni”, is used for sushi. The demand for sea urchin roe has grown as Japanese food increases in popularity in the North American food industry [1]. Sea urchin roe is considered a high quality food product with the price greatly influenced by factors such as appearance, colour, texture, and flavour [2]. *E. chloroticus* is fished off the coasts of New Zealand both commercially and recreationally for its roe. The reported commercial catch of *E. chloroticus* for 2013 was 875,031 kilograms, mainly obtained off the South Coast [3]. The majority of *E. chloroticus* roe is sold in New Zealand as a local delicacy, with some exports sent to Australia [4].

There has been research interest in enhancing the quality and yield of *E. chloroticus* roe to increase the return value for the domestic market. This would also potentially create opportunities for exportation to overseas markets that demand specific qualities in roe [5]. Although most assessments of sea urchin roe quality have previously been on physical differences, recent studies sought to identify protein and metabolite differences contributing to variation in quality for *E. chloroticus* roe, specifically to variations in colour [6]. It was hypothesised from the results that binding proteins targeting carotenoid molecules, the major source of pigmentation in roe, might affect colour. Efforts have thus been made to identify carotenoid binding proteins in the roe [7]. Despite the research interest in *E. chloroticus,* very little genetic information was available for this species in public databases. There was an opportunity to develop genetic data for *E. chloroticus*, which would facilitate a genomic based analysis. For this purpose, we conducted a whole transcriptome sequencing project aiming to characterise transcripts from various tissues. This would provide a source of genetic information to aid current and future research involving *E. chloroticus*.

As well as a valued food product, sea urchins have long been used as a model organism in areas such as developmental and systems biology. Their importance as a research model system for modern molecular, evolutionary, and cell biology led to the genome sequencing project for the sea urchin species *Strongylocentrotus purpuratus*[8]. The genome was estimated to encode around 23,300 genes, and was shown to share many pathways with humans including orthologs to human disease genes. Also discovered was the lack of an adaptive immune system, and instead the possession of a large innate immune system that contained a diverse range of pathogen-binding motifs [8]. The many innate immune proteins encoded in the sea urchin genome are considered a valuable resource for antimicrobial applications and for furthering our understanding of the human innate immune system [9]. Gene structure in the *S. purpuratus* genome has subsequently been further defined by transcriptome analysis [10], but aside from *S. purpuratus* there is little genomic data available for any other sea urchin species from public databases. Genomic data produced for the *E. chloroticus* species would be a novel resource in addition to the *S. purpuratus* data for any research involving the sea urchin as a model system.

*E. chloroticus* is currently placed as the single species of its genus *Evechinus* under the Echinometridae family of sea urchins, which belong to the marine phylum Echinodermata. The Echinometridae family includes species that are geographically close to *E. chloroticus* such as *Heliocidaris* located off the south coast of Australia and *Echinometra* located in the Indo-West Pacific and Pacific to Atlantic oceans. The Echinometridae family had recently been placed in a superfamily called Odontophora with the Strongylocentrotidae and Toxopneustidae families based phylogenetic and morphological data [11]. The placement of *E. chloroticus* with the Echinometridae species had been based on morphological evidence and was described as morphologically close to species from the *Heliocidaris* genus, specifically *H. tuberculata*[12]. The genus *Evechinus*, the following history of which was described by McRae (1958) [12], was first placed in the Echinidae family by H. L. Clark (1912) who later (1925) shifted *Evechinus* and *Heliocidaris* to the Strongylocentrodidae family based on the polyporus ambulacral plates and circular ambitus of *Heliocidaris*, and the larval specialisation and the pedicellariae of *Evechinus*. Their placement was later contended by Mortensen (1943) who moved the genera to their current family Echinometridae based on the strongly developed single lateral tooth of the gemmiform pedicellariae, the paired nature of the poison glands, and the structure of the larval forms [12]. The established relationships of *E. chloroticus* to other sea urchin species has been based solely on morphological evidence, and has yet to be analysed at a genomic level. Obtaining transcriptome data would provide an opportunity to compare the sequence similarity of *E. chloroticus* genes to other sea urchins species and infer new information about its phylogenetic relationships.

Here we describe the extraction of RNA from multiple tissue types of *E. chloroticus* followed by their sequencing using Next Generation Sequence (NGS) technology. Transcripts were reconstructed by *de novo* assembly and annotated by sequence similarity to public protein databases to provide a set of transcripts along with functional annotations. Lastly, we describe the relationship of *E. chloroticus* to other sea urchin species based on the sequence similarity of selected genes.

## Results and discussion

### Sequencing and quality control

Libraries (cDNA) were constructed using RNA extracted from selected tissue samples from a male and female *E. chloroticus*. These animals were taken from Doubtful Sound, Fiordland, New Zealand, and housed feeding on kelp at the New Zealand Marine Studies Centre, Portobello, Dunedin, for 3 years. Tissues used for sequencing included the roe, muscle, gut tissue, water vascular system and also a sample of the coelomic fluid. Samples were harvested, snap frozen and total RNA extracted using a RNAeasy kit with a Qiagen**®** shredder. Equal amounts of total RNA from each tissue sample were combined to give the mixed RNA sample. RNA from an individual male and female, consisting of a mixed total tissue sample, a roe tissue sample and a coelomic fluid sample, were used to generate six libraries.

Sequencing was carried out on an Illumina HiSeq-2000 machine, which generated 123 million pairs of 100 base length paired-end reads (24.8 Gb). The raw sequence data in FASTQ format was submitted to the National Centre for Biotechnology Information (NCBI) Sequence Read Archive (SRA) database and accessible through the BioProject [Accession: PRJNA190637]. Quality control was carried out on the raw reads prior to assembly. This involved the removal of adapter sequences, trimming of low quality bases (Q < 20) from both ends of reads and discarding reads less than 25 bases in length. Reads from potential contaminating species were also removed. Quality control resulted in a total of 118,016,465 high quality reads (95.3% of total raw reads) with 94.5% remaining paired-end. These reads were then used for transcript assembly.

### *De novo* assembly

**Figure 1:**
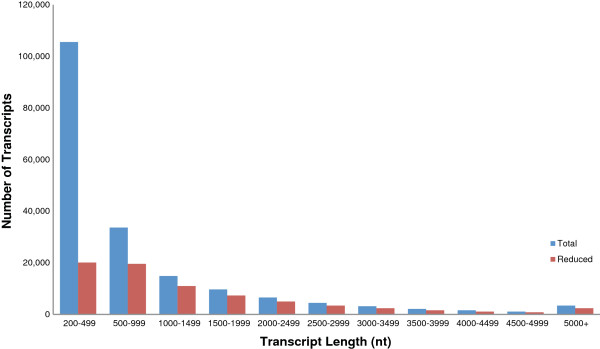
**Length distribution of assembled transcripts for total and reduced datasets.** The sizes of the Trinity assembled transcripts are shown. The reduced set has an FPKM >0.5.

The *de novo* assembly of the quality processed reads using the Trinity assembly program resulted in 209,654 transcript isoforms. Reads used in the assembly were aligned to the assembled transcripts and lowly represented isoform sequences with less than 1% of their gene (component) reads were removed resulting in 186,427 transcripts. This represents the total set of transcripts from the assembly. Transcripts had a total length of 169,963,629 bp (~170 Mb) with a median length of 415 bp, mean length of 912 bp, GC% of 39.0, and a N50 of 1,839 bp (Table 1). A large proportion of the transcripts were short and had a low number of mapped reads. To generate an additional subset transcripts with low coverage were filtered using a minimum FPKM (Fragments Per Kilobase of exon per Million fragments mapped) value of 0.5, which resulted in a reduced set of 75,002 transcripts. This FPKM of 0.5 corresponded to a minimum of 41 reads per kilobase of transcript, which equated to an average base coverage of approximately 8.2. Removing the low abundance transcripts doubled the median length to 923 (Table 1). The main reason for producing a reduced set was for use in the annotation step, shorter transcripts were less likely to be protein coding and able to be annotated, also transcripts less than 200 bp in length cannot be submitted to the NCBI Transcriptome Shotgun Assembly database (TSA). Transcripts in the reduced set had a total length of 106,210,820 bp (~106 Mb) with a median length of 923 bp, mean length of 1,416 bp, GC% of 38.7, and a N50 of 2,242 bp (Table 1). The length distribution of transcripts for the total and reduced sets is shown in Figure 1. As seen in the graph the large reduction in transcripts between the sets after abundance filtering relates to a large removal of short transcripts between 200 to 500 bases in length. Transcripts were submitted to the NCBI TSA database [Accession: GAPB00000000]. The set of reads used for the assembly was aligned to the total and reduced transcript sets for read representation (Table 1). Out of 118 M total reads, 92.2% of the reads mapped to the transcripts in the total transcript set and 91.2% mapped to the reduced set. This showed that the 111,425 transcripts removed from the total set represented only 1% of the total reads.

**Table 1:** Assembly statistics

	**Total**	**Reduced**
Number of transcripts	186,427	75,002
Total transcripts length (bp)	169,963,629	106,210,820
Mean transcript length (bp)	912	1,416
Median transcript length (bp)	415	923
Minimum transcript length (bp)	201	201
Maximum transcript length (bp)	19,251	19,251
N50 (bp)	1,839	2,242
GC %	39.0	38.7
Total read alignment %	92.2	91.2

**Figure 2:**
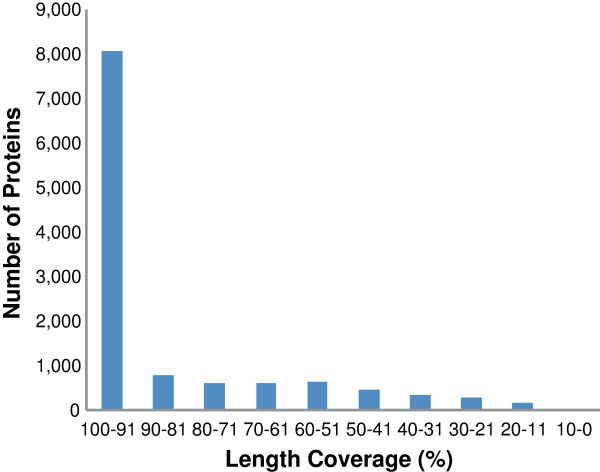
**Protein length coverage against *S. purpuratus *proteins.** Most matches to *S. purpuratus* fall into the top bin (100-91%).

The completeness of the assembly was assessed by two means, the reconstruction of transcripts with full-length proteins and the representation of core conserved genes. To assess the reconstruction of full-length proteins, the 75,002 transcripts were scanned for possible open reading frames (ORFs). A total of 23,870 coded proteins were predicted and tested for similarity to *S. purpuratus* proteins, available from the NCBI protein database, using BLASTP with a cut-off E-value of 10^-20^. A total of 20,974 of the predicted *E. chloroticus* proteins matched to 11,906 unique *S. purpuratus* proteins. For each unique *S. purpuratus* protein the length coverage was calculated using the best matching *E. chloroticus* protein and the distribution of percent length coverage is shown in Figure 2. For the *S. purpuratus* proteins, 8,060 (68%) had a match to an *E. chloroticus* protein with >90% alignment coverage. This shows that a majority of these proteins were near full-length. The euKaryotic clusters of Orthologous Groups (KOGs) from the Clusters of Orthologous Groups (COG) represents a database of protein sequences with conserved domains between eukaryotes. The CEGMA (Core Eukaryotic Genes Mapping Approach) program was used to assess the representation of core eukaryotic proteins in the transcript set. CEGMA uses a representative set of 458 Core Eukaryotic Genes (CEGs) that are highly conserved between eukaryotic species. A total of 453 of the 458 CEGs had a match to a transcript in the reduced set. CEGMA also reports on a subset of 248 most highly conserved CEGs as a measurement of genome completeness. Out of the subset of 248 CEGs there were matches to 246 with 243 being ‘complete’ (more than 70% of protein length aligned) and only 3 being partial. This showed the transcriptome assembly was able to successfully assemble a majority of transcripts for core eukaryotic genes.

### Functional annotation

**Figure 3:**
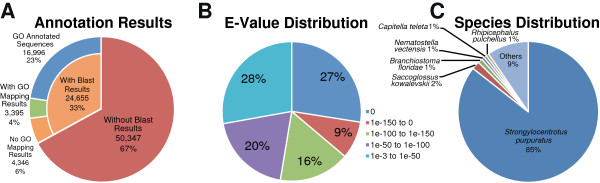
**BLASTX annotation results.** (**A**) Proportions of transcripts with BLASTX matches and GO terms annotated. (**B**) Distribution of E-values for the top hit for each transcript. (**C**) Distribution of species for the top hit for each transcript. Analysis by Blast2GO. The data is available on the accompanying website.

Functional annotation was carried out on the reduced set of 75,002 transcripts. BLASTX searches were performed for each transcript against the NCBI non-redundant protein database and for each result the top 20 hits with E-values less than 10^-3^ were saved. A total of 24,655 of the 75,002 transcripts (33%) had a BLASTX match to a known protein within the database (Figure 3A). The 50,347 transcripts (67%) without a BLASTX result were mostly shorter length sequences (less than 2 kb) that likely do not have a protein coding sequence. However, included in this group though will be transcripts that code for novel proteins without any similar sequence in the database, as well as polyadenylated non-coding RNAs.

The distribution of E-values for the top hits showed that 72% of transcripts had a strong match to a database protein (E < 10^-50^) (Figure 3B). The species distribution for the top hits showed 85% of top hits to proteins from *S. purpuratus* (Figure 3C). Previous research on *S. purpuratus*, including the genome sequencing project, has provided a set of annotated proteins for this sea urchin species. This resource provided the majority of best annotations for the *E. chloroticus* transcripts. The next species most represented in the top hits at 2% was *Saccoglossus kowalevskii. S. kowalevskii* is a species of acorn worm, which are closely related to members of the Echinodermata phylum [13]. Out of the 458 transcripts with protein top hits to *S. kowalevskii*, most appeared to have less significant matches to *S. purpuratus*. The relatively large proportion of *S. kowalevskii* top hits could be due to longer, more complete protein sequence available for *S. kowalevskii* over *S. purpuratus* for those transcripts. The proportion of others (9%) represented all other specific species with top hits to less than 1% of the transcripts and of these 24 transcripts had ‘unknown’ species as their top hit.

**Figure 4:**
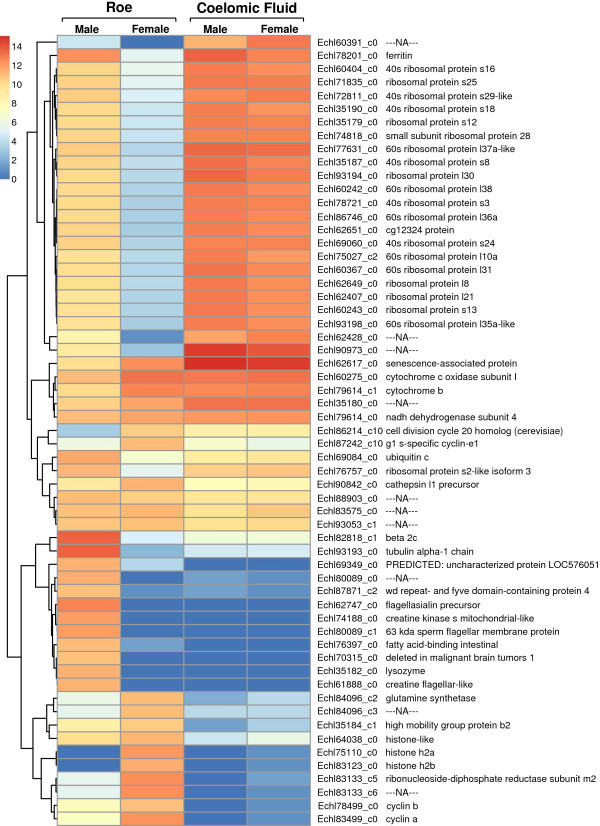
**Heatmap of most highly expressed transcripts from each tissue.** Heatmap showing the log_2_ FPKM values of most abundant transcripts. The colour legend shows the log_2_ FPKM values each represents. Rows were clustered based on Euclidean distance.

The most abundant transcripts within different samples were assessed. Reads were aligned to transcripts to generate FPKM values at a gene level for male roe, female roe, male coelomic fluid and female coelomic fluid samples. The top 20 transcripts with highest FPKM values for each sample can be seen in Additional file 1. The expression levels of each unique top transcript were compared across each sample by taking the log_2_ of the FPKM and clustered based on Euclidean distance. The heatmap produced with these values (Figure 4) showed groups of transcripts where the abundance related to sample type, sex, or both. The most abundant transcripts from coelomic fluid cells were mostly ribosomal proteins, which were found to be relatively expressed less in the roe. There was a similar expression of transcripts from the coelomic fluid between male and female. Ferritin was expressed higher in cells from the coelomic fluid compared to the roe and also higher in the male samples compared to the female samples. Several transcripts were similarly expressed at high levels across all samples, which included essential proteins such as cytochrome b and c, NADH and a senescence-associated protein. Transcripts that were specifically abundant in the male roe included sperm production related proteins, such as the sperm flagellar membrane, flagellasialin precursor and creatine flagellar-like proteins. Levels of tubulin proteins were also higher in male roe. Cell replication related proteins such as histone, cyclin and ribonucleoside-diphosphate reductase were higher in roe samples than coelomic fluids. Female roe specifically had higher levels of these transcripts compared to male.

The number of transcripts with a BLASTX match (24,655) was similar to the number of predicted proteins (23,870). A large portion of the transcripts (50,347) remained without a significant BLASTX match. To analyse these transcripts further we assessed the representation of repeated sequences. Both the reduced set of transcripts and the unannotated set without a BLASTX match were analysed using the RepeatMasker program with the latest Repbase database to identify repeating elements. The summary of RepeatMasker results (Table 2) shows repeating elements that were detected, which included a number of different retroelements and DNA transposons. When these results were compared to that for the *S. purpuratus* genome, obtained from the RepeatMasker site, there were a lot of similarities in the relative counts for repeating elements. SINEs represented the majority of retroelements for *E. chloroticus*, in *S. purpuratus* the majority of retroelements were tRNA-related SINEs. The most represented LINEs for *E. chloroticus* were the L2/CR1/Rex, the L2 was the most represented for *S. purpuratus*. For the LTR elements the Gypsy/DIRS1 were the most represented for both. The most represented DNA transposon for both was PiggyBac, which in *S. purpuratus* is the most abundant repeating element taking up 31% of total repeated sequence (28% in *E. chloroticus*). There were a small number of ncRNA fragments that corresponded mainly to rRNA. The results for the reduced set of transcripts were compared to results from the unannotated set using the percentage of sequence values for relative levels of element abundance. Retroelements were slightly less abundant in the unannotated set (0.80% of total sequence) when compared to the reduced set (1.12%), which was likely due to coded reverse transcriptases annotated by BLASTX. There was a large decrease in LINEs (0.57% to 0.15%) and LTRs (0.19% to 0.05%) in the unannotated set, but an increase in SINE elements (0.36% to 0.59%). Comparing the abundance of DNA transposons there was an increase in the unannotated set (1.14% to 1.98%), which mostly corresponded with the increase in PiggyBac (0.66% to 1.25%). Overall, there was only a small increase in the proportion of total interspersed repeats between the reduced set (2.34%) and the unannotated set (2.90%).

**Table 2:** Summary of repeating elements

	**Reduced set (Unannotated set)**	
**Number of transcripts**	75, 002 (50,347)	
**Total sequence length (MB)**	106 (44)	
	**Number of elements**	**Percentage of total sequence (%)**
**Retroelements**	5,180 (2,922)	1.12 (0.80)
**SINEs:**	3,360 (2,349)	0.36 (0.59)
Penelope	5 (0)	~0 (0)
**LINEs:**	1,398 (421)	0.57 (0.15)
CRE/SLACS	0	0
L2/CR1/Rex	999 (297)	0.40 (0.10)
R1/LOA/Jockey	32 (5)	0.01 (~0)
R2/R4/NeSL	34 (7)	0.01 (~0)
RTE/Bov-B	134 (68)	0.04 (0.02)
L1/CIN4	112 (19)	0.07 (0.01)
**LTR element:**	442 (152)	0.19 (0.05)
BEL/Pao	106 (44)	0.05 (0.01)
Gypsy/DIRS1	314 (96)	0.14 (0.04)
**DNA transposons**	9,363 (6,976)	1.14 (1.98)
Hobo-Activator	566 (403)	0.05 (0.08)
Tc1-IS6030-Pogo	1,305 (865)	0.14 (0.20)
PiggyBac	4,862 (3,853)	0.66 (1.25)
Tourist/Harbinger	86 (55)	0.02 (0.01)
Other (Mirage,P-element,Transib)	1 (1)	~0 (~0)
**Unclassified**	552 (426)	0.08 (0.13)
**ncRNA fragments (mainly rRNA)**	110 (25)	0.02 (~0)
**Total interspersed repeats**		2.34 (2.90)
**Satellites**	19 (0)	~0 (~0)
**Simple repeats**	31,104 (17,306)	1.19 (1.51)
**Low complexity**	6,010 (3,170)	0.32 (0.41)

The redundancy of unannotated transcripts was assessed by clustering transcripts based on 90% or more sequence similarity. Out of 50,347 transcripts, this resulted in 44,687 clusters of non-redundant transcripts. The proportion of nucleotide sequence matches to the unannotated transcripts was assessed by using BLASTN to search against the NCBI non-redundant nucleotide database with a cut-off E-value of 10^-6^. Only 3,254 (6.5%) transcripts had a match to a database nucleotide sequence. These results for the unannotated transcripts showed that a large number of transcripts in the reduced set contained novel sequence without a protein or nucleotide match to the NCBI non-redundant databases. These transcripts are not due to overly redundant sequences or an increased proportion of repeated sequence.

There are challenges to providing a complete annotation of transcripts from a *de novo* assembly. High-throughput annotation approaches such as multiple BLAST searches provide a practical means to giving automated annotations to transcriptome datasets, but the broad nature of this search is limited by the detail and sensitivity of the annotation. From the proportion of unannotated *E. chloroticus* transcripts there will be biologically important transcripts that could not be annotated by this approach.

As an example we looked to identify a non-coding transcript for telomerase RNA. Telomerase RNA is a non-coding RNA that together with the telomerase reverse transcriptase protein forms a ribonucleoprotein enzyme that is essential for synthesising the telomeric DNA repeats at the ends of chromosomes [14]. The first invertebrate telomerase RNA sequence was recently identified using a targeted strategy in *S. purpuratus*[15], which gave an opportunity to attempt to identify this transcript in *E. chloroticus*. A BLASTN search using the 535 bp telomerase RNA sequence from *S. purpuratus* identified an unannotated 520 bp transcript from *E. chloroticus* with an E-value of less than 10^-114^. The *E. chloroticus* and *S. purpuratus* sequences were aligned and conserved domains identified (Additional file 2). The *E. chloroticus* sequence showed close similarity to the *S. purpuratus* sequence with 76% identity. The universal template-pseudoknot domain and the vertebrate specific H/ACA domain could be identified. However, while the Box H and CAB box could be identified, the terminal Box ACA motif was not covered, suggesting that the end of the *E. chloroticus* assembly was truncated. While a number of transcripts could not be annotated with multiple BLASTX/BLASTN searches, this unannotated set still contains biologically important transcripts, such as the telomerase RNA, that can be discovered through such a detailed search.

### Gene ontology (GO) annotation

**Figure 5:**
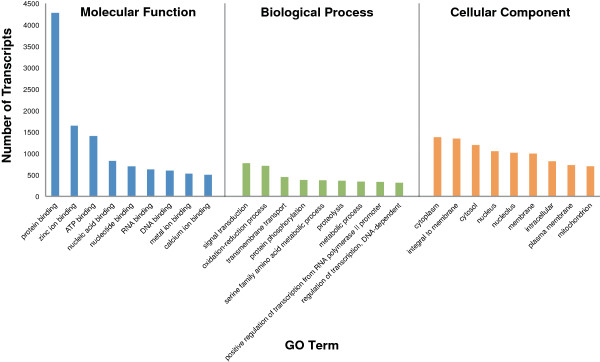
**GO annotations.** Top 10 represented GO terms for each of the GO categories; Molecular Function, Biological Process and Cellular Component. GO terms annotated by protein similarity to the NCBI non-redundant database and from InterProScan results.

Gene Ontology (GO) terms were assigned to transcripts through Blast2GO. All GO terms associated with the BLASTX matches were retrieved and mapped to each transcript. Scores were computed for each GO term and the most specific terms used to annotate the transcript. Out of the 24,655 transcripts with BLASTX results, 20,309 transcripts had GO mapping results with 16,914 receiving GO annotations. Additional GO terms could be annotated to transcripts using InterPro protein domains assigned through InterProScan. An additional 82 transcripts with no BLASTX result received GO annotations through associated protein domains (Figure 3A). The top most represented GO terms for each of the three GO categories, molecular function, biological process and cellular component, are shown in Figure 5. The top represented GO terms for molecular function were from binding domains; protein binding (4,275) followed by zinc ion binding (1,646) and ATP binding (1,401). For biological process, the top GO terms were signal transduction (773) followed by oxidation-reduction process (711) and transmembrane transport (451). For cellular component, the top GO terms were cytoplasm (1,379) followed by integral to membrane (1,348) and cytosol (1,198).

### InterPro annotation

**Figure 6:**
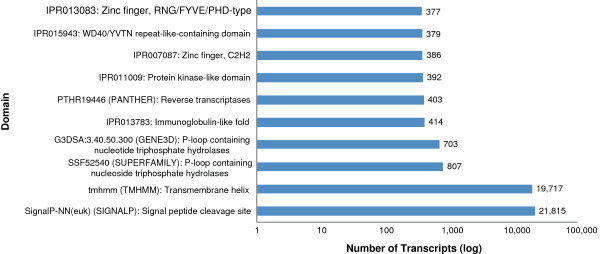
**InterPro annotations.** Top 10 represented InterPro terms from the InterProScan annotations.

InterProScan was used to search with the longest ORF from each transcript against the InterPro database, an integrated database of protein domains and functional sites. This provided additional annotation based on conserved structural domains; 40,573 transcripts received InterPro annotation. Additional GO terms could be retrieved from the InterProScan results and merged with existing GO annotations, 11,478 transcripts had GO terms associated with InterPro results. The most represented features (Figure 6) were signal peptide cleavage sites (21,815) and the transmembrane helix domains (14,717), followed by the P-loop containing nucleotide triphosphate hydrolases superfamily (807 from SUPERFAMILY and 703 from GENE3D databases). The next highest represented was the immunoglobulin-like fold (414) and the reverse transcriptases family (403). The genome sequencing of *S. purpuratus* revealed that sea urchins have a large innate immune system with many pathogen recognition genes, providing an opportunity to use the sea urchin model in areas of evolutionary immunobiology [16]. The Toll-like receptor family has expanded in sea urchins; the *S. purpuratus* genome contains 253 Toll-like receptors (IPR000157, PF01582), whereas the human genome contains only 11. There were 45 matches within the *E. chloroticus* transcripts to a Toll-like receptor domain, and proteins from these Toll-like receptor transcripts were predicted to be mostly full length (median percentage coverage of 80% to *S. purpuratus* proteins). This new transcript data will be useful in studies on the sea urchin immune system.

### EC and KEGG Orthology annotations

**Figure 7:**
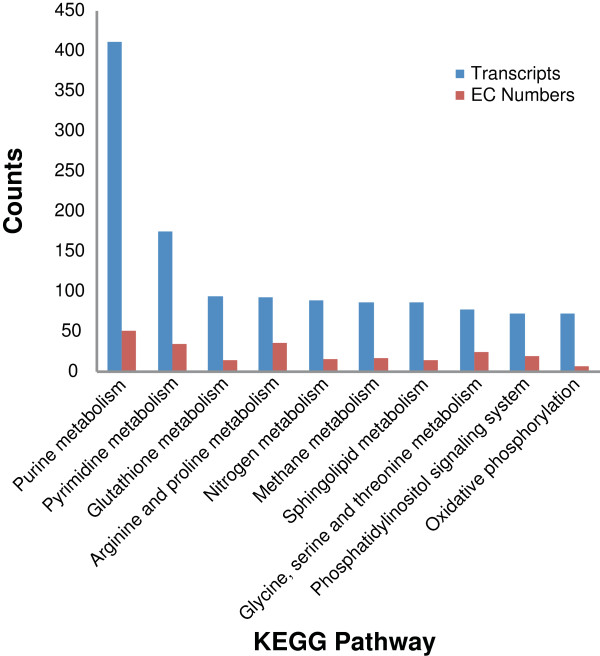
**KEGG pathway annotations.** Top 10 represented KEGG pathways using EC (Enzyme Commission) annotations, derived from GO annotations. Shown is the number of transcripts matching to each pathway and the number of EC numbers represented in the pathways.

**Figure 8:**
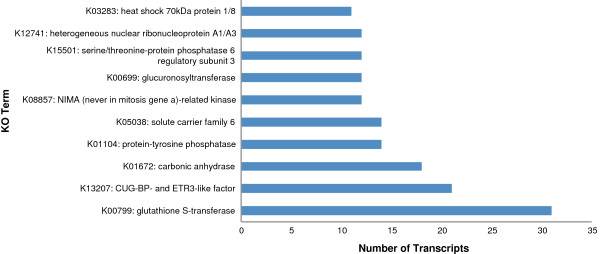
**KO annotations.** Top 10 represented KO terms from the KAAS annotation results.

Using the annotated GO terms associated EC (Enzyme Commission) numbers could be assigned to the transcripts. A total of 4,227 transcripts were assigned 1,087 unique EC numbers, which were across 127 different KEGG pathways; the top represented pathways are shown in Figure 7. The top 8 pathways were related to metabolism; the most represented was the purine metabolism pathway with 411 transcripts across 51 enzymes, followed by pyrimidine metabolism with 175 transcripts across 34 enzymes and glutathione metabolism with 94 transcripts across 15 enzymes. The transcripts were also analysed using the KEGG Automatic Annotation Server (KAAS) to provide annotations of KEGG Orthology (KO) codes, resulting in 7,173 transcripts being annotated to 4,213 unique KOs. The most represented KOs are shown in Figure 8.

### Phylogenetic analysis

**Figure 9:**
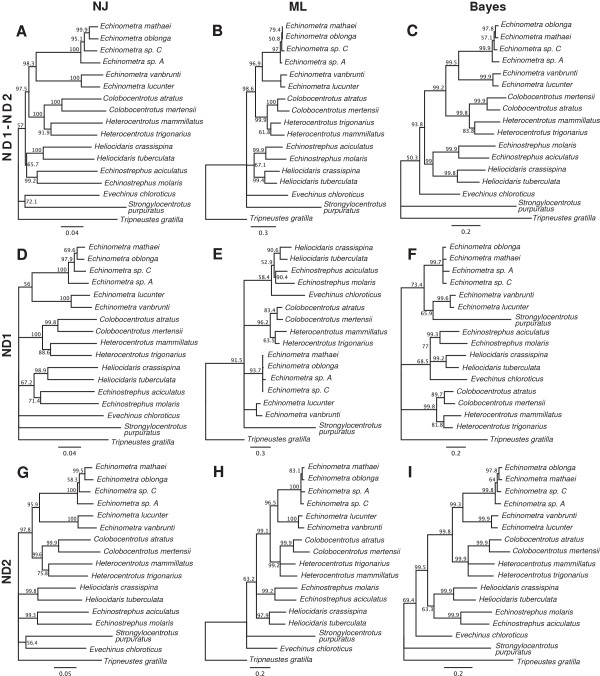
**Phylogenetic trees.** Resulting trees for the phylogenetic analysis of Echinometridae species based on the nucleotide sequences of ND1, ND2 and combined ND1-ND2 genes. Trees were constructed using neighbour-joining (NJ), maximum-likelihood (ML), and Bayesian inference (Bayes) methods. Numbers at each internal node represent the bootstrap percentage values for the nodes support. The scale bar represents base substitutions per site. Shown are the: ND1-ND2 NJ (**A**), ML (**B**), Bayes (**C**) trees; ND1 NJ (**D**), ML (**E**), Bayes (**F**) trees; and ND2 NJ (**G**), ML (**H**), Bayes (**I**) trees.

Previously, the phylogenetic relationship of *E. chloroticus* to other sea urchin species had not been analysed. The genetic data produced from this study allowed a first look at the phylogenetic relationship of *E. chloroticus* to other members of its family. There are few molecular studies on the phylogeny of the Echinometridae family. A study by Kinjo *et al.*, (2008) [17] used the mitochondrial DNA phylogeny of Echinometridae to deduce the evolutionary history of their larval skeletal morphology. They compared the mitochondrial NADH dehydrogenase genes ND1 and ND2 of 14 Echinometridae species that represented a majority of the genera. They constructed phylogenetic trees for the ND1, ND2 and ND1-ND2 combined sequences using neighbour-joining (NJ), maximum-likelihood (ML) and maximum parsimony methods. Using the combination of the slower mutagenic ND1 and faster mutagenic ND2, their ND1-ND2 phylogenetic tree grouped each genus into a monophyletic clade and grouped the Echinometridae species together from two outgroup species, which were from the two sister families Strongylocentrotidae and Toxopneustidae. Groupings also formed between different genera giving insight into the relationships within the Echinometridae family. To analyse the relationship of *E. chloroticus* to other Echinometridae species the sequence data from the study by Kinjo *et al.*, (2008) for the ND1 and ND2 mitochondrial genes was used for a phylogenetic analysis with the addition of the *E. chloroticus* genes. Phylogenetic trees were constructed for the ND1, ND2, and ND1-ND2 combined sequences using NJ, ML and Bayesian inference (Bayes) methods (Figure 9) with supporting values of bootstrap percentages.

The resulting trees were very consistent with those from Kinjo *et al.*, (2008). The ND1-ND2 trees (Figure 9A-C) supported the monophyly of each genus and the relationships between genera reported in Kinjo *et al.*, (2008) were represented in these trees. The grouping of all Echinometridae species into a single clade from the two outgroup species was not supported for the ML tree, but was achieved with low support for the NJ tree and higher support for the Bayes tree. Analysing the placement of *E. chloroticus* in the ND1-ND2 trees, *E. chloroticus* did not form any clades with any of the other Echinometridae species. The ML tree placed *E. chloroticus* in the same position as the outgroup species *S. purpuratus*, separate from the Echinometridae clades formed. The NJ tree placed *E. chloroticus* outside of the Echinometridae family clade and in a clade with *S. purpuratus*. The Bayes tree, which had the highest supporting values for each Echinometridae clade, placed *E. chloroticus* outside of a highly supported family level clade for Echinometridae. These results showed *E. chloroticus* is phylogenetically distinct from the other species of its family.

Kinjo *et al.* (2008) described ND2 as having higher rates of mutation compared to other mitochondrial genes and therefore useful for determining close relationships between species. The ND1 had a slower rate of mutation and was described as being useful for determining distant relationships. In the separate ND2 trees (Figure 9G-I) *E. chloroticus* was placed in the same positions as in the ND1-ND2 trees. The ND2 sequence has therefore been significant in constructing the ND1-ND2 trees and the faster mutating gene could not determine any relationships between *E. chloroticus* and the other Echinometridae. In the ND1 trees (Figure 9D-F) *E. chloroticus* was placed in a clade with the two genera clades for *Heliocidaris* and *Echinostrephus* with low support in the ML tree and higher support in the Bayes tree. The Bayes tree though unexpectedly placed *S. purpuratus* as a sister to one of the *Echinometra* clades. This result may have been the ability of the ND1 gene to detect distant relationships as these species belonged to the same superfamily. The results from the ND1 ML and Bayes trees suggested a distant relationship between *E. chloroticus* and the *Heliocidaris* and *Echinostrephus* genera.

The results of the phylogenetic comparison of these two mitochondrial genes showed *E. chloroticus* as a distinct species that had significant genetic difference to other species of its family. This greater difference in genetic sequence in *E. chloroticus* compared with that of other Echinometridae species may be attributed to the fact that *E. chloroticus* had lived in isolation, solely around the coasts of New Zealand, for millions of years since its speciation [18]. The geographical isolation of *E. chloroticus* would have prevented genetic exchange through fertilisation with other Echinometridae. Mutations over time would cause genetic differences to build up within the *E. chloroticus* species, and due to its isolation they would remain unique, unable to be shared with other Echinometridae. This could have led to the early speciation of *E. chloroticus*. The other species of Echinometridae remained in contact longer and could have taken longer to speciate and become genetically different from each other through unique mutations. The earlier speciation of *E. chloroticus* would explain the greater number of genetic differences in its gene sequences compared to other Echinometridae species, which led to its distinct place in the phylogenetic trees. Although separated, *E. chloroticus* remained morphologically similar to other Echinometridae species. This could be due to the similar environments the species share, specifically with the *Heliocidaris* species off the southern coasts of Australia, and there had not been pressures for greater morphological difference. *E. chloroticus* therefore had remained morphologically similar to species within its family, but had become significantly different phylogenetically due to its early separation and long period of isolation around New Zealand. A comprehensive phylogenetic analysis of the Echinometridae family is limited by the range of species for which genomic data is available. Such a study would be valuable in uncovering the distant relationships between species within this family and to predict the time of the geographical isolation of *E. chloroticus*.

## Conclusions

We report the first genome wide dataset for the New Zealand sea urchin species *E. chloroticus*. High-throughput sequencing of RNA extracted from multiple tissue samples followed by *de novo* transcriptome assembly has produced a dataset of 75,002 transcripts for *E. chloroticus*. A total of 24,655 transcripts had a significant protein match to the NCBI non-redundant database. Transcripts were annotated with GO terms, InterPro domains and KO terms to provide additional functional information. This new data allowed the first phylogenetic analysis of *E. chloroticus* to other species of its family Echinometridae. Comparison of ND1 and ND2 sequence revealed *E. chloroticus* as being genetically distinct from its family, as it did not form any strong clades with other family members and remained outside of family level clades. There is an opportunity to further analyse the phylogenetic relationship of *E. chloroticus* to other sea urchin species and uncover its evolutionary history. This transcriptome data will provide a valuable resource of genomic information for this unique sea urchin species for studies either looking to improve the commercialisation of *E. chloroticus* or using the sea urchin as a model system. Additional datafiles including transcript datasets and functional annotation data are available from the project site: http://mRNA.otago.ac.nz/Kina/.

## Methods

### Sample collection and RNA extraction

*E. chloroticus* specimens were collected from Doubtful Sound, Fiordland, New Zealand. The animals were housed at the New Zealand Marine Studies Centre, Portobello, Dunedin, for 3 years and feed a natural diet of kelp (*Macrocystis pyrifera*). Tissues were harvested from a male and female individual and snap frozen using liquid nitrogen. Using an RNAeasy kit with a Qiagen**®** shredder RNA was extracted from tissues which included the roe, muscle, gut and the water vascular system and also from a sample of the coelomic fluid (containing total coelomocyte cell components). Equal concentrations of RNA were combined from all five sample types to produce a mixed RNA sample. Six RNA samples of equal concentrations consisted of a male mix, roe and coelomic fluid sample, along with a female mix, roe and coelomic fluid sample. Samples with high RNA quality (an RNA Integrity Number (RIN) of over 6) were processed for library construction. *E. chloroticus* is an endemic species and is of particular interest to southern Maori. Consultation with Maori included the University of Otago Ngai Tahu Consultation Committee.

### Sequencing and quality control

Libraries (cDNA) were constructed for the six samples using the Illumina TruSeq RNA protocol. Sequencing was done on an Illumina HiSeq-2000 sequencer, generating 100 base paired-end reads. Reads saved in FASTQ format were quality assessed using FASTQC v0.10.1 (http://www.bioinformatics.babraham.ac.uk/projects/fastqc) for base quality and adapter sequence. Adapter sequences detected were trimmed from read ends using FastqMcf from ea-utils v1.1.2 (https://code.google.com/p/ea-utils). Bases with low quality phred scores were trimmed from either ends of reads using DynamicTrim with a phred score cut-off of 20. Length filtering of reads was performed using LengthSort with a minimal length of 25 bases. Both DynamicTrim and LengthSort are part of the SolexaQA package v2.2 (http://solexaqa.sourceforge.net) [19]. Bowtie2 v2.1.0 (http://bowtie-bio.sourceforge.net/bowtie2) [20] was used to align reads to genomic sequences of possible contaminating species (e.g. *Homo sapiens*). Potential contaminating reads were then aligned back to sea urchin genomic sequence, retaining any likely *E. chloroticus* reads.

### *De novo* assembly

*De novo* assembly of the processed reads into transcripts was carried out using the Trinity assembly program release 2013-02-25 (http://trinityrnaseq.sourceforge.net) [21] using default parameters. Minimum length for reported transcripts was 200 bases. To assess transcript abundance paired reads were aligned to the transcripts through a Trinity script, which used Bowtie v0.12.9 (http://bowtie-bio.sourceforge.net) [22] to align reads. RSEM v1.2.3 (http://deweylab.biostat.wisc.edu/rsem) was then used to generate isoform percentages and FPKM values. Transcripts of the same trinity component could be treated as different sequence isoforms. Transcripts that had less than 1% of the total reads for their component were considered unsupported isoforms and removed from the set. Transcripts were then filtered based on a minimal FPKM value of 0.5 which corresponded to 41 reads per kilobase to give a reduced set of transcripts. This was to remove transcripts from the assembly that had relatively low read support and were therefore less likely to be complete. The majority of transcripts removed were short, being less than 500 bases in length. The reduced set was then selected for functional annotation. Transcript statistics were computed using in-house scripts. Bowtie2 v2.1.0 was used to align all reads used in the assembly to the total and reduced transcript sets for read representation statistics. Trinity scripts were used to generate the protein coverage statistics by extracting predicted *E. chloroticus* proteins from transcript ORFs, followed by BLASTP [23] searches to *S. purpuratus* proteins obtained from the NCBI database. The CEGMA v2.4 program (http://korflab.ucdavis.edu/datasets/cegma) [24] was used to provide KOG annotations of transcripts and reports for completeness of Core Eukaryotic Genes.

### Functional annotation

The Blast2GO v2.6.6 program (http://www.blast2go.com) [25] was used extensively for the functional annotation of transcripts. BLASTX [23] searches were used within Blast2GO to the NCBI non-redundant protein database to identify similar proteins using an E-value cut-off of 10^-3^ and the top 20 hits for each transcript were recorded. The top hit for each transcript was selected for E-value and species distributions. Transcript abundance was calculated by aligning reads from each of the tissue samples to the transcripts using Bowtie2 v2.1.0 followed by RSEM for FPKM values. Using the Trinity transcript component level for gene distinction, the top 20 genes from each tissue sample with the highest FPKM values were selected to show expression levels of the most abundant transcripts. The pheatmap package v0.7.4 [26] within R v3.0.1 [27] was used to create a heatmap using the log_2_ of FPKM values of each top gene across each tissue. Rows were clustered by Euclidean distance. The RepeatMasker v2.2.27+ (http://www.repeatmasker.org) program was used along with the latest (22-04-2013) Repbase database (http://www.girinst.org/repbase) [28] to identify repeating elements. Default settings were used and the query species was set as Echinoidea. RepeatMasker used a Smith-Waterman-Gotoh type algorithm to search for matching sequences to known interspersed repeats (retroelements, DNA transposons), simple repeats (microsatellites), and fragments of ncRNAs, that were represented in the Repbase database. Low complexity sequences (polypurine, polypyrimidine, and regions of extremely high AT (>87%) or GC (>89%) content) were also identified with RepeatMasker. Clustering of transcripts was performed using CD-HIT-EST (http://weizhong-lab.ucsd.edu/cd-hit) [29] at 90% similarity to assess transcript redundancy. For nucleotide sequence matches, BLASTN [23] searches were used to the NCBI non-redundant nucleotide database using an E-value cut-off of 10^-6^. An increased E-value cut-off was used to provide more unique and meaningful nucleotide matches from the BLASTN search. *S. purpuratus* nucleotide sequence [Accession: JQ684708] was used in a local BLASTN [23] search to identify an *E. chloroticus* telomerase RNA transcript and the sequences were aligned in Geneious v6.1.4 program (http://www.geneious.com) using the Geneious aligner. GO annotations were assigned to transcripts by Blast2GO using the BLASTX results. InterProScan [30] searches were used within Blast2GO to InterPro databases [31] to provide InterPro annotations of conserved protein domains and functional sites. Blast2GO then used InterPro results to add additional GO annotations to transcripts based on associated terms. Enzyme codes were assigned to transcripts by Blast2GO based on associated GO terms providing results for EC numbers and their KEGG pathways. The KEGG Automatic Annotation Server (KAAS) (http://www.genome.jp/tools/kaas) [32] was used to annotate transcripts with KO codes.

### Phylogenetic analysis

For the phylogenetic analysis of *E. chloroticus* and the Echinometridae species, the ND1 and ND2 sequence data used in Kinjo *et al.*, (2008) [17] was downloaded from the NCBI database [Accession: AB178488 - AB178518]. A total of 14 species from the Echinometridae family were used, which included the six *Echinometra, E. sp. A*, *E. sp. C*, *E. lucunter*, *E. mathaei*, *E. oblonga*, *E. vanbrunti*; the two *Colobocentrotus*, *C. atratus*, *C. mertensii*; the two *Heterocentrotus*, *H. mammillatus*, *H. trigonarius*; the two *Echinostrephus*, *E. aciculatus*, *E. molaris*; and the two *Heliocidaris*, *H. crassispina*, *H. tuberculata*. The two outgroup species used were *Tripneustes gratilla* from the sister family *Toxopneustidae* and *S. purpuratus* from the sister family *Strongylocentrotidae*. Sequence data for *S. purpuratus* was obtained from NCBI [Accession: NC_001453]. The *E. chloroticus* ND1 and ND2 sequence was obtained by a BLASTN search with the *S. purpuratus* genes against the assembled transcripts. The *E. chloroticus* mitochondrial genome was almost fully reconstructed across several transcripts. The transcript identified containing the ND1 and ND2 genes was aligned with all the previous sequences and 819 matching bases for ND1 and 1059 matching bases for ND2 were extracted for the following analysis. The phylogenetic trees were constructed within the Geneious v6.1.4 program. ND1, ND2, as well as combined ND1-ND2 sequences from each species were aligned to each other. All alignments were carried out using the Geneious Aligner within Geneious, set at 70% identity. The NJ trees were constructed with the Geneious Tree Builder using the HKY85 model [33] with 1000 bootstrap repeats. The ML trees were constructed with the PhyML [34] Geneious module using the HKY85 model with 1000 bootstrap repeats and estimated gamma distribution and invariable site proportion. The Bayes trees were constructed with the MrBayes [35] Geneious module using the HKY85 model with gamma distribution and invariable site estimations, gamma categories of 4, chain length of 50,000, heated chains at 4, subsampling frequency of 10, and a burn-in length of 1000. All trees were set at a bootstrap threshold of 50% and rooted using *T. gratilla* as the outgroup.

## Competing interests

The authors declare that they have no competing interests.

## Authors’ contributions

GG carried out the bioinformatic analysis of the sequence data and drafted the manuscript. DG contributed to sample collection, RNA extraction and manuscript revision. CB proposed and supervised the study and contributed to the manuscript. All authors have read and approved the final version of the manuscript.

## Supplementary Material

Additional file 1 **Top expressed transcripts in tissue samples.** Table of the top 20 transcripts with the highest FPKM values for four different tissue types; male roe, female roe, male coelomic fluid and female coelomic fluid. Click here for file

Additional file 2 ***E. chloroticus *** **telomerase RNA sequence alignment.** Sequence alignment of *E. chloroticus* telomerase RNA to *S. purpuratus* telomerase RNA was performed in the Geneious program using the Genious aligner. Conserved sequence is highlighted black. Structural domains are labelled for the template-pseudoknot (purple) and H/ACA (blue) domains. Click here for file
