# Supplementary material for: The transcriptome of the NZ endemic sea urchin Kina (Evechinus chloroticus)
Source: BMC Genomics. 2014 Jan 20;15:45. doi: 10.1186/1471-2164-15-45 (PMC3898728; doi:10.1186/1471-2164-15-45)
Supplement: Additional file 1 — Top expressed transcripts in tissue samples. Table of the top 20 transcripts with the highest FPKM values for four different tissue types; male roe, female roe, male coelomic fluid and female coelomic fluid. [file 1471-2164-15-45-S1.docx]

| **Additional file 1 Top expressed transcripts in tissue samples** | | | |
| --- | --- | --- | --- |
| **Transcript ID** | **Mean length (bp)** | **FPKM** | **Annotation** |
| **Male roe** | | | |
| Echl93193_c0 | 1,786 | 13,336 | tubulin alpha-1 chain |
| Echl82818_c1 | 1,802 | 12,865 | beta 2c |
| Echl62747_c0 | 1,073 | 6,998 | flagellasialin precursor |
| Echl78201_c0 | 1,255 | 5,691 | ferritin |
| Echl74188_c0 | 2,229 | 4,137 | creatine kinase s- mitochondrial-like |
| Echl80089_c1 | 1,358 | 3,959 | 63 kda sperm flagellar membrane protein |
| Echl69084_c0 | 413 | 3,285 | ubiquitin c |
| Echl80089_c0 | 312 | 3,009 | ---NA--- |
| Echl35182_c0 | 1,225 | 2,803 | lysozyme |
| Echl61888_c0 | 5,101 | 2,674 | creatine flagellar-like |
| Echl69349_c0 | 1,786 | 2,585 | PREDICTED: uncharacterized protein LOC576051 |
| Echl76757_c0 | 2,237 | 2,335 | ribosomal protein s2-like isoform 3 |
| Echl79614_c0 | 4,590 | 2,314 | nadh dehydrogenase subunit 4 |
| Echl88903_c0 | 347 | 2,306 | ---NA--- |
| Echl76397_c0 | 1,013 | 2,126 | fatty acid-binding intestinal |
| Echl60275_c0 | 6,206 | 2,103 | cytochrome c oxidase subunit I |
| Echl70315_c0 | 2,589 | 2,019 | deleted in malignant brain tumors 1 |
| Echl87871_c2 | 1,698 | 1,904 | wd repeat- and fyve domain-containing protein 4 |
| Echl83575_c0 | 512 | 1,889 | ---NA--- |
| Echl93053_c1 | 343 | 1,868 | ---NA--- |
| **Female roe** | | | |
| Echl60275_c0 | 6,206 | 9,132 | cytochrome c oxidase subunit I |
| Echl79614_c1 | 3,689 | 7,281 | cytochrome b |
| Echl62617_c0 | 7,044 | 5,701 | senescence-associated protein |
| Echl83133_c6 | 873 | 5,650 | ---NA--- |
| Echl83133_c5 | 2,343 | 5,377 | ribonucleoside-diphosphate reductase subunit m2 |
| Echl83499_c0 | 4,601 | 5,095 | cyclin a |
| Echl75110_c0 | 2,195 | 4,534 | histone h2a |
| Echl79614_c0 | 4,590 | 3,594 | nadh dehydrogenase subunit 4 |
| Echl35180_c0 | 1,219 | 3,534 | ---NA--- |
| Echl83123_c0 | 2,685 | 3,128 | histone h2b |
| Echl90842_c0 | 1,410 | 2,692 | cathepsin l1 precursor |
| Echl83575_c0 | 512 | 2,533 | ---NA--- |
| Echl64038_c0 | 1,779 | 2,334 | histone -like |
| Echl84096_c2 | 1,855 | 2,316 | glutamine synthetase |
| Echl87242_c10 | 1,580 | 2,268 | g1 s-specific cyclin-e1 |
| Echl84096_c3 | 982 | 2,125 | ---NA--- |
| Echl78499_c0 | 4,058 | 1,942 | cyclin b |
| Echl93053_c1 | 343 | 1,893 | ---NA--- |
| Echl35184_c1 | 2,370 | 1,591 | high mobility group protein b2 |
| Echl86214_c10 | 1,544 | 1,567 | cell division cycle 20 homolog (cerevisiae) |
| **Male coelomic fluid** | | | |
| Echl62617_c0 | 7,044 | 35,249 | senescence-associated protein |
| Echl90973_c0 | 1,571 | 26,425 | ---NA--- |
| Echl78201_c0 | 1,255 | 13,123 | ferritin |
| Echl93194_c0 | 489 | 11,516 | ribosomal protein l30 |
| Echl77631_c0 | 639 | 11,413 | 60s ribosomal protein l37a-like |
| Echl35187_c0 | 734 | 10,064 | 40s ribosomal protein s8 |
| Echl60367_c0 | 477 | 9,275 | 60s ribosomal protein l31 |
| Echl35180_c0 | 1,219 | 9,245 | ---NA--- |
| Echl60275_c0 | 6,206 | 9,010 | cytochrome c oxidase subunit I |
| Echl71835_c0 | 494 | 8,823 | ribosomal protein s25 |
| Echl60243_c0 | 585 | 8,521 | ribosomal protein s13 |
| Echl60242_c0 | 375 | 8,507 | 60s ribosomal protein l38 |
| Echl62407_c0 | 698 | 8,411 | ribosomal protein l21 |
| Echl62649_c0 | 851 | 8,347 | ribosomal protein l8 |
| Echl93198_c0 | 489 | 8,016 | 60s ribosomal protein l35a-like |
| Echl86746_c0 | 719 | 7,918 | 60s ribosomal protein l36a |
| Echl60404_c0 | 726 | 7,855 | 40s ribosomal protein s16 |
| Echl78721_c0 | 977 | 7,815 | 40s ribosomal protein s3 |
| Echl75027_c2 | 360 | 7,733 | 60s ribosomal protein l10a |
| Echl35190_c0 | 606 | 7,506 | 40s ribosomal protein s18 |
| **Female coelomic fluid** | | | |
| Echl62617_c0 | 7,044 | 26,494 | senescence-associated protein |
| Echl90973_c0 | 1,571 | 15,725 | ---NA--- |
| Echl77631_c0 | 639 | 10,328 | 60s ribosomal protein l37a-like |
| Echl60275_c0 | 6,206 | 9,992 | cytochrome c oxidase subunit I |
| Echl35180_c0 | 1,219 | 9,589 | ---NA--- |
| Echl60391_c0 | 302 | 8,763 | ---NA--- |
| Echl72811_c0 | 565 | 8,073 | 40s ribosomal protein s29-like |
| Echl71835_c0 | 494 | 7,443 | ribosomal protein s25 |
| Echl93194_c0 | 489 | 7,336 | ribosomal protein l30 |
| Echl79614_c1 | 3,689 | 7,221 | cytochrome b |
| Echl62428_c0 | 319 | 7,166 | ---NA--- |
| Echl35179_c0 | 708 | 7,130 | ribosomal protein s12 |
| Echl69060_c0 | 574 | 7,095 | 40s ribosomal protein s24 |
| Echl74818_c0 | 509 | 6,861 | small subunit ribosomal protein 28 |
| Echl35187_c0 | 734 | 6,849 | 40s ribosomal protein s8 |
| Echl78721_c0 | 977 | 6,698 | 40s ribosomal protein s3 |
| Echl78201_c0 | 1,255 | 6,665 | ferritin |
| Echl60367_c0 | 477 | 6,580 | 60s ribosomal protein l31 |
| Echl62651_c0 | 504 | 6,429 | cg12324 protein |
| Echl86746_c0 | 719 | 6,326 | 60s ribosomal protein l36a |
