# Supplementary figures and images for: The transcriptome of the NZ endemic sea urchin Kina (Evechinus chloroticus)
Source: BMC Genomics. 2014 Jan 20;15:45. doi: 10.1186/1471-2164-15-45 (PMC3898728; doi:10.1186/1471-2164-15-45)

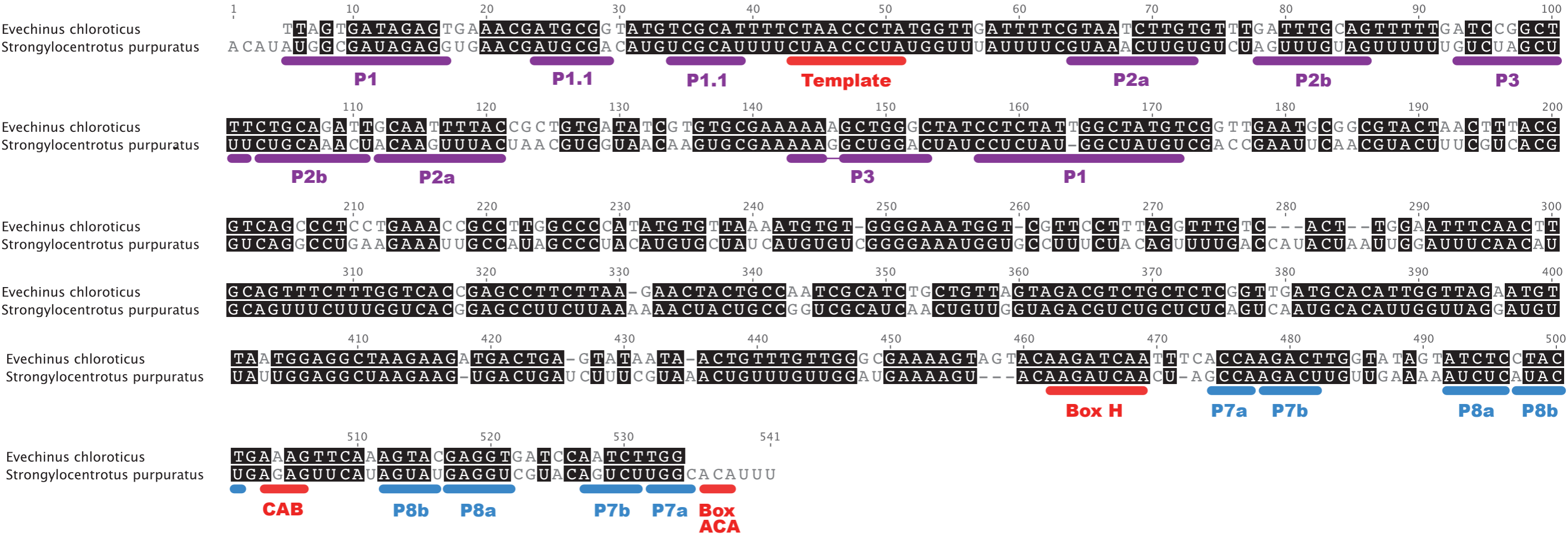

Supplement: Additional file 2 — E. chloroticus telomerase RNA sequence alignment. Sequence alignment of E. chloroticus telomerase RNA to S. purpuratus telomerase RNA was performed in the Geneious program using the Genious aligner. Conserved sequence is highlighted black. Structural domains are labelled for the template-pseudoknot (purple) and H/ACA (blue) domains. [file 1471-2164-15-45-S2.pdf]
